# Safety Monitoring in Group A Meningococcal Conjugate Vaccine Trials: Description, Challenges, and Lessons

**DOI:** 10.1093/cid/civ509

**Published:** 2015-11-09

**Authors:** Godwin C. Enwere, Gandhali Paranjape, Prasad S. Kulkarni, Manisha Ginde, Katharina Hartmann, Simonetta Viviani, Julie Chaumont, Lionel Martellet, Marie-Francoise Makadi, Karen Ivinson, Elisa Marchetti, Jacques Herve, Kim Kertson, F. Marc LaForce, Marie-Pierre Preziosi

**Affiliations:** 1 Meningitis Vaccine Project, PATH, Ferney-Voltaire, France; 2 DiagnoSearch Laboratories, Mumbai,; 3 Serum Institute of India, Ltd, Pune; 4 Department of Pharmacovigilance and Pharmacoepidemiology, Eidgenössische Technische Hochschule, Zürich; 5 Meningitis Vaccine Project, Department of Immunization, Vaccines and Biologicals, World Health Organization, Geneva, Switzerland

**Keywords:** MenAfriVac, safety monitoring and reporting, uniform methods, effective communication

## Abstract

***Background.*** The determination of the safety profile of any vaccine is critical to its widespread use in any population. In addition, the application of international guidelines to fit local context could be a challenging but important step toward obtaining quality safety data.

***Methods.*** In clinical studies of PsA-TT (MenAfriVac), safety was monitored immediately after vaccination, at 4–7 days for postimmunization local and systemic reactions, within 28 days for adverse events, and throughout the duration of study for serious adverse events. Initial and ongoing training of sites' staff were undertaken during the studies, and a data and safety monitoring board reviewed all the data during and after the studies.

***Results.*** The safety of PsA-TT was evaluated according to international standards despite obvious challenges in remote areas where these studies were conducted. These challenges included the need for uniformity of methods, timely reporting in the context of frequent communication problems, occurrence of seasonal diseases such as malaria and rotavirus diarrhea, and healthcare systems that required improvement.

***Conclusions.*** The trials of PsA-TT highlighted the value of a robust vaccine development plan and design so that lessons learned in initial studies were incorporated into the subsequent ones, initial training and periodic retraining, strict monitoring of all procedures, and continuous channel of communication with all stakeholders that enabled the application of international requirements to local settings, with high quality of data.

Determination of the safety profile of any vaccine or drug is crucial before its routine utilization in the community, as drugs that show a safe profile in animal studies can show a different effect in humans [1]. Appropriate study designs and study sizes are needed to determine both short- and long-term safety [2]. In the 1990s, the use of high-dose measles vaccine was associated with increased mortality in females, a finding that led the World Health Organization (WHO) to rescind its earlier recommendation on the vaccine [3, 4]. Although scientists scrambled to explain the reason for such an unusual finding, it further emphasized the need for due diligence in assessing the safety of any vaccine before it can be introduced into the population [3, 5]. There was a similar occurrence with the first oral rotavirus vaccine [6].

Following the introduction of *Haemophilus influenzae* type b (Hib) conjugate, there were reports of occurrence of Guillain-Barré syndrome (GBS) [7, 8]. In a rather more dramatic fashion, rare cases of GBS were also reported in the United States in a temporal relationship following administration of Menactra [9]. Although several hypotheses have been propounded to explain the possible occurrence of GBS following immunization [8, 10], comparison with expected rates of GBS has been inconclusive to define if there is increased risk of GBS with the administration of diphtheria, tetanus, polio, Hib combination vaccine, and quadrivalent meningococcal conjugate vaccine [10, 11]. Taken together, these findings highlight the need for due rigor in establishment of the safety profile of any vaccine. Thus, the trials for PsA-TT (MenAfriVac) were designed to ensure that both common and rare adverse events (AEs) were detectable throughout the clinical development period.

## DESIGN OF SAFETY COMPONENT OF VACCINE TRIALS AND RATIONALE

Eight clinical trials were conducted in groups ranging from 14 weeks to 29 years of age, in 5 countries (India, Gambia, Mali, Senegal, and Ghana) during the clinical development of PsA-TT. These trials were double-blind randomized controlled studies designed to allow adequate comparison of the safety profile of PsA-TT with that of 2 licensed vaccines; some of the results have been reported elsewhere [12].

Group A *Neisseria meningitidis* infections occur across various regions and cultures with heterogeneous characteristics. In the design of the safety component of the trials of PsA-TT, uniform system and harmonized procedures were used to collect the data, while making allowance for peculiarities in each of the settings where the studies were done. This was necessary to ease combination of the data sets and comparison of specific characteristics between groups as the trials took place in several areas with cultural and geographic variations. These trials also complied with the Good Clinical Practice (GCP) standard and other international regulatory requirements.

## DESCRIPTION OF METHODS OF SAFETY ASSESSMENT

Across all the trials, safety assessments were done at specified time points as per standard operating procedures (SOPs) from the sponsors and the sites.

### Immediate Safety Assessment

The purpose of this was to detect any adverse reactions that occur immediately after administration of the study vaccine. Subjects were observed before the vaccination and then after vaccination for between 30 minutes and 60 minutes. During this period, subjects were assessed for any AE or any immediate hypersensitivity reaction. The injection site was also examined for any local reaction such as induration or tenderness.

### Postimmunization Local and Systemic Reaction Monitoring

Participants were visited daily for 4 days (7 days in the initial studies) to document any systemic or local reaction. The follow-up on day 4 (or 7) was done by a medical doctor. A consistent method of soliciting systemic reactions was used, and the participants or their caregivers were asked nonleading questions. Any solicited systemic reactions were recorded, and the injection site was examined to document any local reaction such as tenderness and induration. Study staff assessed the intensity of the tenderness using a priori–defined grading criteria. The size of any induration and temperature were also measured.

### Recording of Adverse Events

Untoward medical occurrences were recorded from the date of vaccine administration until 28 days following vaccination. The diagnosis, date of onset, intensity, and treatment given were recorded. As much as possible, these AEs were defined in terms of diagnosis, but if this was not possible, symptomatic AEs were documented. Medical doctors assessed the causality of AEs based on priori–defined criteria (see below).

### Recording of Serious Adverse Events

As per International Conference on Harmonisation (ICH) guidelines [13], a serious adverse event (SAE) was defined as an untoward medical occurrence that results in death, is life threatening, results in persistent or significant disability/incapacity, requires inpatient hospitalization or prolongation of existing hospitalization, is a congenital anomaly/birth defect in the offspring of a study subject, or is an important medical event that may jeopardize the subject or may require intervention to prevent one of the other serious outcomes. These were identified and documented by the investigators, who were responsible for appropriate care for the subjects and timely reporting of the SAEs. The recording of SAEs started immediately, when the subject was enrolled in the study, and continued until the end of the study. The intensity of any SAE was assessed using predefined criteria; the treatment given and the outcome of the event were documented in the case report form.

## ASSESSMENT OF CAUSALITY

Before the start of the PsA-TT studies, it was decided that any solicited event elicited within the first 4 (or 7) days after vaccination would be regarded as an adverse reaction—that is, they would be assumed to be caused by study vaccines. This decision eases the difficulty that might occur in assigning causality immediately after the study vaccine was given, but is mindful of the fact that many may not be related to the study vaccine. The assessment of causality for other AEs or SAEs was based on 3 defined considerations: the temporal relationship of the event to the time the study vaccine was given; the possibility of other obvious causes; and the previous AE experience of the study vaccine or other similar conjugate vaccines as specified in the Investigator's Brochure. In all PsA-TT trials, conforming to current practice in clinical development, only 2 grades were used for the assessment of causality: related and unrelated.

## TRAINING

A very important component of the AE and SAE monitoring was the rigor taken in training all those involved in the studies. Trainings included basic GCP training, training on the protocol and SOPs, and practically verified training on procedures. Nurses and field workers received bedside training on identification of common clinical signs, assessment of intensity, and measurement of dimensions of local reactions. Physicians were also trained to ensure uniformity in the ascertainment of clinical signs and assigning causality. In some studies, parents and subjects were trained to document AEs. These trainings were done at the beginning of each study, and at specified milestones—for instance, after recruitment of a specified percentage of the sample size. There were also in-built, ongoing, on-the-job trainings through periodic monthly or fortnightly business meetings and person-to-person interaction. Data on error rate and common errors were regularly generated at the study sites to facilitate these trainings.

## REPORTING OF SERIOUS ADVERSE EVENTS

**Figure 1.**
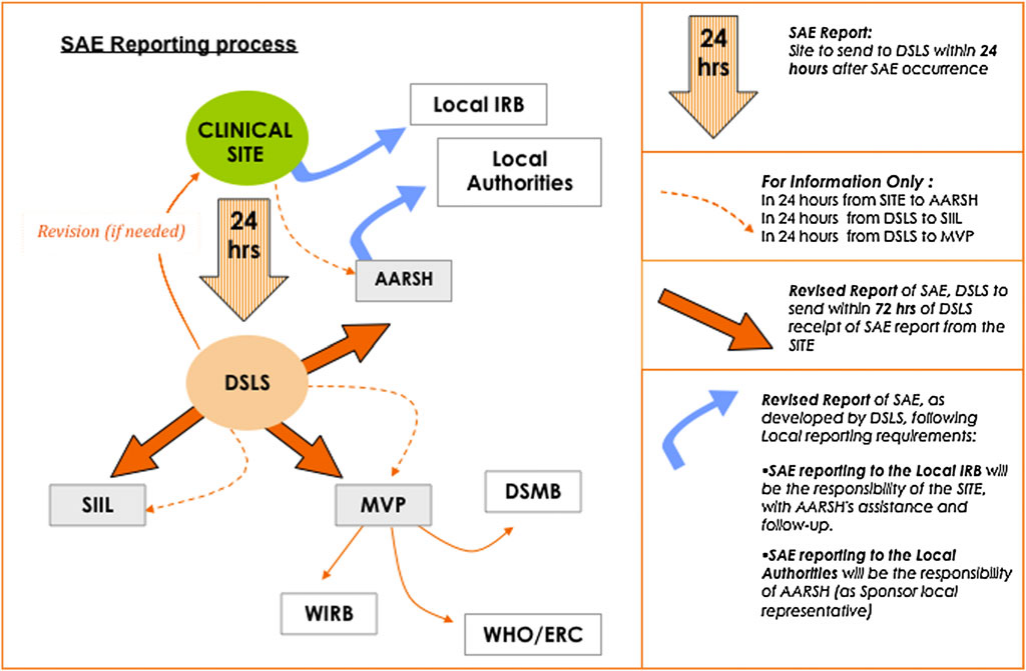
Adverse events and serious adverse events (SAE) reporting system for PsA-TT trials (MenAfriVac). Abbreviations: AARSH, Agence Africaine de Recherche en Santé Humaine (monitors); DSLS, DiagnoSearch Life Sciences (pharmacovigilance coordination); DSMB, data and safety monitoring board; IRB, institutional review board; MVP, Meningitis Vaccine Project; SIIL, Serum Institute of India, Ltd. (vaccine manufacturers); WHO/ERC, World Health Organization Ethic Review Committee; WIRB, Western Institute Review Board.

A simple reporting system of AEs and SAEs (Figure 1) was employed in the PsA-TT clinical trials. Investigators reported all SAEs to the Contract Research Organization (CRO), which reported to the sponsors. The sponsors (or their agent) in turn reported the SAEs to the data and safety monitoring board (DSMB) and various ethics review committees and regulatory authorities. The investigators also reported the SAEs to the local ethics review committees. As the study progressed, there was an accumulation of experiences and innovation that helped to simplify the system. At the site, specific staff was designated to be in charge of all SAE communications. This helped to decrease errors and ensured that each SAE was properly documented, tracked, and followed up in accordance with specified timelines.

During the earlier phases of the PsA-TT trials, SAEs were reported with handwritten forms. As the studies progressed, to comply with the Council for International Organizations of Medical Sciences reporting system [14], recording of AE data on an electronic template was introduced. This made the process more efficient, and any new information was highlighted to ease identification. Designated staff was put in charge of this and was responsible for numbering the follow-up SAE reports and tracking log.

## MONITORING

### Overall Monitoring Scheme

To ensure the collection of valid data, there was a culture of monitoring from the field sites up to the level of the DSMB. Investigators monitored one another to ensure the accuracy, completeness of AE forms, and also timeliness of the SAE reporting. SAE reports were checked for accuracy and compliance with the protocol by the responsible CRO. A different CRO performed periodic monitoring visits to the study sites, where they compared the source documents with what was recorded on the SAE report forms and/or case report forms, to reconcile any discrepancy. The frequency of the visits was determined by the nature of the study and the quantity of data to be monitored. The sponsors also employed the services of an experienced safety consultant who helped set up the SAE system and during the course of studies clarified terms and important dates and resolved differences in understanding.

### Role of DSMB

A DSMB of 3–4 experts was set up prior to initiation of each PsA-TT clinical trial in accordance with GCP requirements and the DSMB charter. These comprised distinguished epidemiologists, pediatricians, and a respected lay member of the community. For each study, there was a mixture of international and local experts. The group conducted face-to-face meetings or telephone conferences, and thoroughly assessed the data during the course of each study, providing the sponsors with overall review of the study to ensure adequate protection of study subjects. The board reviewed SAE reports and all AEs at specified intervals and advised the sponsors on any concerns in relation to the vaccine safety, in addition to making appropriate recommendations. Important safety-related activities or study participants’ health issues were reviewed with the DSMB, and the advice was taken into consideration in decisions related to these issues.

### Role of Periodic Meetings

Early on in the clinical development, it was noted that understanding of issues and SOP items varied across trial sites. This was not surprising, considering the wide cultural differences in the different places where the studies took place. Efforts were made to harmonize these differences so as not to jeopardize subjects’ health. Periodic meetings were set up between the sites and sponsors; sites and CRO; and sites, CRO, and sponsors to discuss any misunderstanding, misinterpretation, and challenges to ensure a common understanding and practice. When necessary, further trainings were organized at the sites to improve the understanding and implementation of specific aspects of the SOP. Also during the course of each trial, meetings were set up to clarify issues such as SAE onset date, end date, and any issue with the reporting system. Each of the different collaborators could trigger a meeting, although sponsors bore the overall responsibility for ensuring that safety activities in the trials were done to international standards.

## CHALLENGES

### Need for Uniformity

Because harmonized understanding and ascertainment procedures for safety assessment were crucial in view of diverse cultural, linguistic, and religious environments, extra efforts were made to meet this standard. Much effort was invested in ensuring uniform translations of the study documents and understanding by study workers. Close monitoring of the data collected helped to detect misunderstanding (eg, between “swelling” and “induration”), which was tackled by further training, demonstration, and the development of a field manual for a “ballpoint” measuring technique [15]. Similarly, an issue was observed in one of the study sites in relation to defining the onset date of SAEs and a hospital stay that fulfilled the inpatient hospitalization criterion. At this site, subjects preferred to be treated as outpatients rather than being admitted, and others wanted to be put on parenteral medication even when the doctor did not think this necessary. The SOP for this site was revised to accommodate this peculiar environment. Periodic meetings were held to ensure that the safety of subjects was not jeopardized. Relevant dates were clearly defined.

It is common practice in some of our study sites to give children antipyretics before or following immunization. As this may have an impact on the immune responses following immunization [16, 17], effort was made to document this, and the analytical plan provided for a comparison between those who received and those who did not receive antipyretics.

### Timely Reporting

The studies were performed in rural, suburban, and urban environments with varying communication challenges such as unreliable email network, phone outage, and time differences. There were in-built plans to communicate both internally for the study and with external collaborators and agencies. SOPs clearly specified what to do depending on the circumstance. Study staff used telephone to communicate with subjects or their parent/guardian, and for reporting of AEs to responsible staff. Responsible staff used either email or fax or telephone to report SAEs to the CRO, the sponsors, or the relevant review boards. When email or fax did not work, SAE reports were first communicated by telephone, and the copy was sent as soon as the appropriate communication line was reestablished. Timelines were followed, and any lateness was addressed by the designated coordinator. To ensure that timelines could be kept, minimum information for initial reporting was required as defined by ICH GCP; this included subject identity, the reporter, time of vaccination, date, and description of event with preliminary assessment of severity and causality. Follow-up reports were generated when new information became available. To ensure effectiveness, timelines for conclusions of SAE reports were specified, and every effort was made to stick to these timelines. Lists were generated regularly as a reminder for pending SAE reports and/or summary SAE narratives.

### Differences in Ethical and Regulatory Reporting Requirements

PsA-TT clinical trials had to comply with different approval and reporting systems in sub-Saharan African countries and India in line with GCP. Approval was sought from various regulatory bodies, local ethics review committees, and institutional review boards of partner institutions. As expected, the requirements for these committees varied, and the studies had to work hard to harmonize diversities. For instance, although some committees were satisfied with monthly summary reports, some committees requested an expedited reporting of all SAEs, whether related or not related to the study vaccine, in addition to the monthly summary reports. This unusual directive was particularly challenging to the study site, more so when receipt of most of these transmissions was not acknowledged by the regulatory body. However, every effort was made to comply with them.

### Health Systems in Different Study Sites

PsA-TT clinical trials were conducted in rural areas and cities with varying degrees of functional health systems. One of the important questions in conduct of clinical trials in developing countries is what type of medical care should be given to study subjects [16]. Should this be according to the local medical practice or according to the best international practice? In most rural areas, there were staffing challenges, and various levels of inadequacies including partially functional laboratory facilities. Case ascertainment of events thus required appropriate training across sites to ensure uniformity. Moreover, country-specific treatment policies are tailored according to each country's socioeconomic environment. Medical monitors had to resolve conflicts arising from what they knew regarding normal practice in the local environment. Copies of these policies were obtained for references, and they became the yardsticks to assess what was appropriate for any given case.

In some study sites, a peculiar health system operated that had the use of traditional medications, homeopathic therapy, and orthodox drugs accepted officially. Due to the confidence that many of people from developing countries place in traditional medicines, at times it was difficult to control their use or even to know what medicine a subject actually received. In certain situations, different types of therapy were given together to a study subject. In other situations, the subjects took these themselves, even after receiving the drugs prescribed by study staff. All possible efforts were made to document this and to code with WHO Drug Dictionary.

### Seasonal Diseases and Epidemics Not Related to the Study Disease

The so-called African meningitis belt, which stretches from Dakar in the west to Ethiopia in the east, is also an endemic malaria environment [18]. Malaria is seasonal, and during this period there were increased numbers of SAEs due to malaria, especially in the studies that involved infants and toddlers. Although a priori criteria stipulated that subjects with significant ill health were temporarily exempted from receiving any vaccine, at times it was difficult to diagnose accurately as clinical malaria can be both sudden and dramatic. Although the laboratory confirmation of malaria in most cases helped to distinguish it from the vaccine AEs, in some cases, this was difficult.

At a site where one of the infant studies was conducted, October to February is a known rotavirus diarrhea season. As rotavirus is the commonest cause of diarrhea in infants, there were a number of SAEs of acute watery diarrhea. Fortunately, during the same period, there was ongoing rotavirus surveillance, and this helped to confirm rotavirus infection in most of these cases.

### Safety Evaluation for the Future

Pregnant and lactating women constitute an important group in the community. According to ICH guidelines, pregnant women should be excluded from clinical trials where the drug is not intended for use in pregnancy [13]. The guidance also states that excretion of drug or its metabolites should be monitored in breast milk and infants should be monitored for effects of drugs. These guidelines are issued due to the vulnerability of the developing fetus and infants to the effect of drugs, which is often complex. However in real life, many drugs are prescribed to pregnant women and lactating mothers when the benefit of the drug far outweighs the risk of possible adverse effects. In the PsA-TT trials, none of the studies were designed to assess the safety of the vaccine in pregnant and nursing mothers, and women of childbearing age were advised to use adequate contraception. However, when some subjects inadvertently became pregnant, they were monitored until delivery, and any events in the mother or the newborn were documented. Because women of childbearing age are susceptible to group A meningococcal disease, this raised a dilemma. Consequently, a detailed risk-benefit analysis was conducted, based on burden and impact of the disease in pregnant women, data from nonclinical studies, and experience with similar vaccines, which justified the use of the vaccine in pregnant women during the postlicensure use of the vaccine [19].

PsA-TT (MenAfriVac) has now been given postlicensure to >217 million people aged 1–29 years [20]; hence, women of childbearing age constitute a significant proportion. A study follows up the safety of MenAfriVac in pregnant women and pregnancy outcomes, and data confirm that the vaccine has no adverse effect on pregnant women and their infants [21].

## CONCLUSIONS

Rigorous procedures as required by GCP standards were followed in all the clinical studies to monitor the safety of PsA-TT as well as to ensure safety of the study participants. The following key lessons were learned in the process: (1) It is important to have a robust vaccine development plan and design so that lessons learned in initial studies can be incorporated into subsequent studies; (2) initial training, but crucially also periodic retraining, of all study staff on the protocol and the SOPs is important; (3) strict monitoring of procedures, documentation, and reporting are very beneficial to evaluate the safety of a new vaccine; and (4) a continuous channel of communication with all stakeholders enables the application of international requirements to local settings, with appropriate quality.
